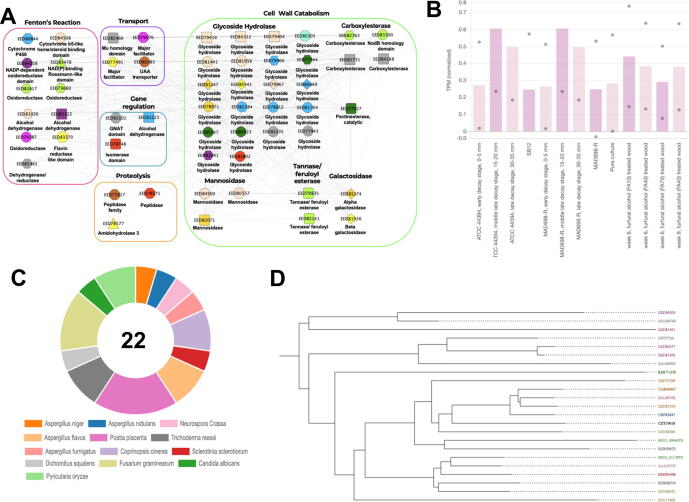# Fungi.guru: Comparative genomic and transcriptomic resource for the fungi kingdom

**DOI:** 10.1016/j.csbj.2020.11.019

**Published:** 2020-11-20

**Authors:** Jolyn Jia Jia Lim, Jace Koh, Jia Rong Moo, Erielle Marie Fajardo Villanueva, Dhira Anindya Putri, Yuen Shan Lim, Wei Song Seetoh, Sriya Mulupuri, Janice Wan Zhen Ng, Nhi Le Uyen Nguyen, Rinta Reji, Herman Foo, Margaret Xuan Zhao, Tong Ling Chan, Edbert Edric Rodrigues, Ryanjit Singh Kairon, Ker Min Hee, Natasha Cassandra Chee, Ann Don Low, Zoe Hui Xin Chen, Shan Chun Lim, Vanessa Lunardi, Tuck Choy Fong, Cherlyn Xin'Er Chua, Kenny Ting Sween Koh, Irene Julca, Riccardo Delli-Ponti, Jonathan Wei Xiong Ng, Marek Mutwil

**Affiliations:** a School of Biological Sciences, Nanyang Technological University, 60 Nanyang Drive, Singapore 637551, Singapore; b College of Medicine and Veterinary Medicine, University of Edinburgh, Old College, South Bridge, Edinburgh EH8 9YL, United Kingdom

**Keywords:** Fungi, Co-expression, Expression, Metabolism, Gene function, Database

## Abstract

The fungi kingdom is composed of eukaryotic heterotrophs, which are responsible for balancing the ecosystem and play a major role as decomposers. They also produce a vast diversity of secondary metabolites, which have antibiotic or pharmacological properties. However, our lack of knowledge of gene function in fungi precludes us from tailoring them to our needs and tapping into their metabolic diversity. To help remedy this, we gathered genomic and gene expression data of 19 most widely-researched fungi to build an online tool, fungi.guru, which contains tools for cross-species identification of conserved pathways, functional gene modules, and gene families. We exemplify how our tool can elucidate the molecular function, biological process and cellular component of genes involved in various biological processes, by identifying a secondary metabolite pathway producing gliotoxin in *Aspergillus fumigatus*, the catabolic pathway of cellulose in *Coprinopsis cinerea* and the conserved DNA replication pathway in *Fusarium graminearum* and *Pyricularia oryzae*. The tool is available at www.fungi.guru.

## Graphical abstract

**Figure:**
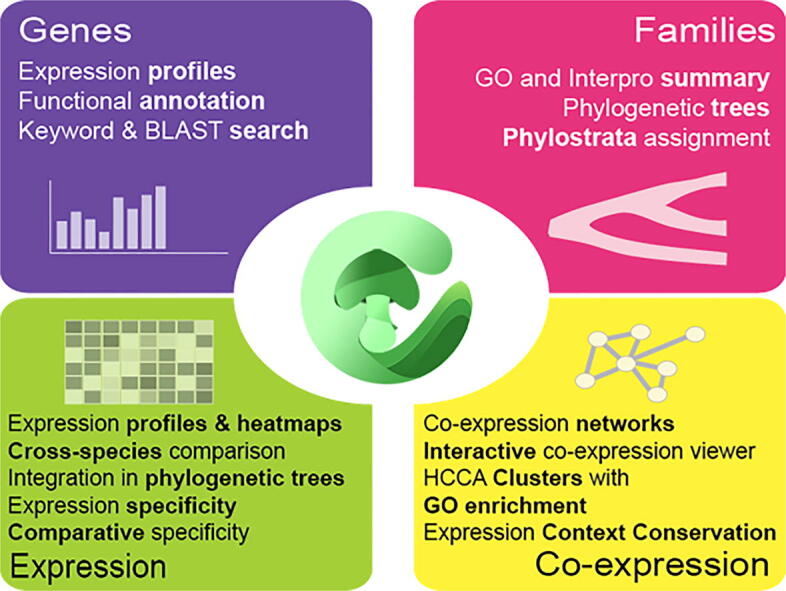

## 1 Introduction

The fungi kingdom comprises an enormous diversity of taxa with varied life cycle strategies, ecologies, and morphologies ranging from unicellular yeasts to large mushrooms. Since ancient times, fungi were used as fermenters and food, but recent mycological research demonstrates yet more novel applications of fungi in food production [1], [2]. While fungi can cause diseases and decay of building materials and fibers [3], fungi have immensely contributed to agriculture and medicine. For example, secondary metabolites naturally produced by Penicillium mould are vital antibiotics such as penicillin [4], while in agriculture, fungi could become a sustainable alternative for animal feed and revolutionize food processing [5]. The sheer diversity of fungal species, estimated at 2.2 to 3.8 million [6], holds much promise for future research, and many fungal genomes and genomic tools are made available in public databases, such as JGI (https://mycocosm.jgi.doe.gov/mycocosm/home)[7], FungiDB (https://fungidb.org/fungidb/)[8], FFGED (http://bioinfo.townsend.yale.edu/)[9], PHI-nets (www.phi-base.org) [10], FPD (http://bis.zju.edu.cn/FPD/) [11], FgMutantDb (https://scabusa.org/FgMutantDb) [12] and eFG (http://csb.shu.edu.cn/efg/) [13]. While these resources are invaluable, none of them provide a large-scale gene expression analyses, precluding us from using this valuable information to study gene function.

As more fungi are being discovered and studied, it is becoming clear how little we know about the gene functions in this kingdom [14]. Genome sequence alone often cannot reveal the function of the species-specific genes, or how the genes work together to form a biological pathway [15]. Genes with similar expression profiles across environmental perturbations, developmental stages, and organs tend to be functionally related, and the identification of these co-expressed genes is thus a powerful tool to study gene function [16], [17], [18], [19], [20], [21] and regulation [22]. These co-expressed genes can be represented as networks, where nodes (or vertices) correspond to genes and edges (or links) connect and indicate genes that have similar expression profiles [23], [24]. The analysis of gene expression data and coexpression networks can reveal groups of functionally related genes (i.e., gene modules), while conservation of these networks over large phylogenetic distances can reveal core components of biological processes [17], [25], [26], [27], [28]. Thus, coexpression analysis is an invaluable tool to reveal gene function [29], but this resource is missing for fungi.

To facilitate the elucidation of gene functions in fungi, we present fungi.guru. This online tool allows analysis of coexpression networks, gene expression profiles, ontology terms, gene families, and conserved coexpression clusters of 19 fungal species. The tool provides a plethora of tools that allow functional inferences of uncharacterized fungal genes. Our tool opens up new possibilities to study fungi and to harness their rich metabolomic arsenal to generate novel drugs and other high-value compounds.

## 2 Materials and methods

### 2.1 Used genomic and transcriptomic data

Publicly available RNA sequencing experiments for the 20 unicellular and multicellular fungi were identified through NCBI SRA [30]. The SRA runtables obtained included experiments from different organs (for multicellular fungi), gene knockout variants, and samples subjected to various chemical and environmental treatments. Coding sequence (CDS) files for each fungus were downloaded from Ensembl Fungi (Table S1) and used to build Kallisto index files for subsequent gene expression estimation with Kallisto v0.46.1 [31]. RNA-sequencing experiments for all the fungal species were streamed as fastq files from the European Nucleotide Archive (ENA) [32] by using the LSTRaP-Cloud pipeline [33]. In total, 40,843 samples comprising 40.8 terabytes were downloaded for 20 fungi (Table S2). TPM (transcripts per million) gene expression values were generated from fastq files by running the Kallisto quant command in the LSTRaP-cloud pipeline with default parameters [33]. For paired-end reads, only the files containing the first read designated with “_1” were downloaded. Single-end and paired-end libraries were mapped with an estimated fragment length of 200 bp and an estimated standard deviation of 20.

To annotate the RNA-seq samples, we used information provided within the SRA runtable and annotation of the sequencing experiments. Annotations include the type of medium the samples were cultivated in, organs, location, sexual/asexual types, temperature, and strains. The annotations were used to construct expression profiles on the CoNekT database [34].

### 2.2 Quality control of RNA-seq experiments and construction of coexpression networks

**Supplementary figure 1:**
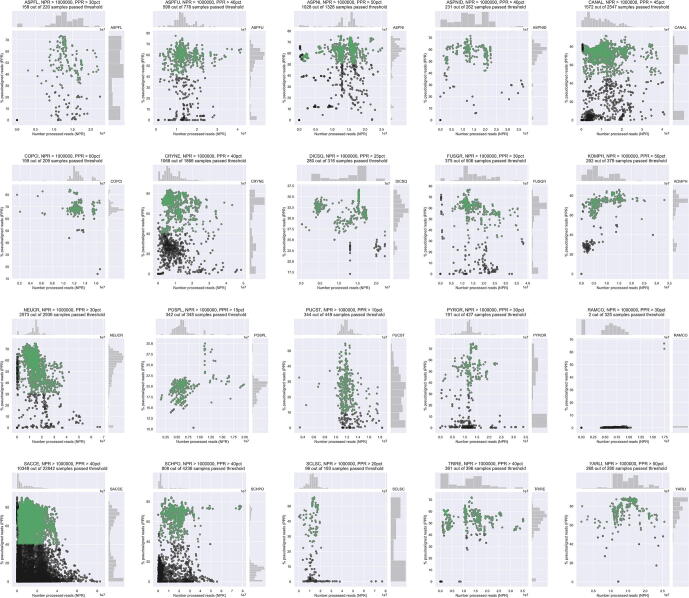


For each fungal species, RNA-seq samples were quality-controlled by removing samples that showed low absolute number of processed reads (NPR) and low percentage of pseudoaligned reads (PPR) to the CDS (denoted by Kallisto as n_pseudoaligned and p_psuedoaligned respectively)[33], [35]. The thresholds were set to remove samples with < 1 million NPR and a species-specific threshold of PPR. The PPR threshold was estimated by manual inspection of scatter plots, which show NPR and PPR values on the x- and y-axis, respectively (Fig. S1). Fungal species *Ramularia collo-cygni* showed low pseudoalignment values and was eliminated from the project. RNA-sequencing samples that passed the thresholds were used to construct expression matrices for each species. In total, 21,135 out of 40,843 samples passed the quality control (Table 1, Table S2).

**Table 1:** Fungal species included in the database.

Species	3-letter code	Division	Use	# samples passed QC / all samples
*Aspergillus flavus*	Afl	Ascomycota	Study of preharvest and postharvest infections, as well as the production of compounds that are toxic to mammals	158 / 220
*Aspergillus fumigatus*	Afu	Ascomycota	Study of invasive aspergillosis (IA), which results in high mortality in AIDS and organ transplant patients	599 / 778
*Aspergillus nidulans*	Asn	Ascomycota	Model organism for studying eukaryotic cell processes such as DNA repair and cell cycle control	1028 / 1328
*Aspergillus niger*	Ani	Ascomycota	Study of black mold, which is a common contaminant of certain fruits and vegetables	1028 / 1328
*Candida albicans*	Cal	Ascomycota	Model organism for fungal pathogens and the study of HIV	1572 / 2547
*Coprinopsis cinerea*	Cci	Basidiomycota	Model organism for studying fungal sex and mating types	199 / 209
*Cryptococcus neoformans*	Crn	Basidiomycota	Model organism for encapsulated fungal organisms and the study of diseases in immunocompromised hosts	1068 / 1866
*Dichomitus squalens*	Dsq	Basidiomycota	Model organism for wood-degrading white-rot fungi that infect both softwood and hardwood plant species	280 / 316
*Fusarium graminearum*	Fgr	Ascomycota	Study of fusarium head blight, a devastating disease affecting wheat and barley	375 / 506
*Komagataella phaffii*	Kph	Ascomycota	Model organism for genetic study and the study of protein production	292 / 379
*Neurospora crassa*	Ncr	Ascomycota	Elucidation of molecular events involved in circadian rhythms, epigenetics and gene silencing, cell polarity, cell fusion and development	2573 / 2936
*Postia placenta*	Ppl	Basidiomycota	Model organism for studying various patterns of wood decay caused by fungi	342 / 348
*Puccinia striiformis*	Pst	Basidiomycota	Study of the effect of stripe rust on wheat and grasses, as well as their potential resistance to stripe rust	344 / 449
*Pyricularia oryzae*	Por	Ascomycota	Study of rice blast, the most important disease concerning the rice crop in the world	191 / 427
*Ramularia collo-cygni*	Rco	Ascomycota	Study of Ramularia leaf spot, a disease affecting barley fields throughout temperate regions worldwide	6 / 330
*Saccharomyces cerevisiae*	Sce	Ascomycota	Model organism for unicellular eukaryotes	10,348 / 22,842
*Schizosaccharomyces pombe*	Sch	Ascomycota	Study of cellular responses to DNA damage and the process of DNA replication	808 / 4236
*Sclerotinia sclerotiorum*	Ssc	Ascomycota	Study of white mold, which is known to infect 408 different plant species	96 / 193
*Trichoderma reesei*	Tre	Ascomycota	Study of cellulase production and cellulose hydrolysis	361 / 396
*Yarrowia lipolytica*	Yli	Ascomycota	Study of the synthesis of valuable metabolites and enzymes, as well as the production of specialty lipids	268 / 280

### 2.3 Functional annotation of fungal coding sequences

The protein IDs were obtained from the pep files available on Ensembl Fungi. Interproscan-5.44–79 [36] was used to obtain the Pfam domains and Gene Ontology terms (GO terms) for each protein in the 20 species. The groups of orthologous genes were obtained using Orthofinder v2.3.12 [37] using Diamond [38] with default settings. Note that the gene trees present in fungi.guru are build using FastTree algorithm, which does not provide bootstrap values. For more confident interpretations of the gene trees, the users are advised to further analyze the sequences of gene families of interest by a more comprehensive phylogenetic analysis, as e.g., provided by the MEGA software (https://www.megasoftware.net/).

### 2.4 Construction of fungi.guru database

The coexpression networks were constructed by using the Highest Reciprocal Rank metric [39]. CoNekT database framework with default settings was used to construct the tool by populating it with the fungal data [34]. Coexpression clusters for each species were generated via Heuristic Cluster Chiseling Algorithm (HCCA)[39], where cluster sizes were limited to 100 genes.

## 3 Results

To allow comparison of the coexpression relationships across the fungi kingdom, the genes have been assigned to Pfam domains (via InterproScan) [36] and gene families (via OrthoFinder) [37]. Our tool offers multiple ways to query it, that can be found in the ‘Search’ panel on the top of the page. The user can find the gene of interest by using gene IDs (e.g., *CBF69394*), sequence similarity (BLAST), Pfam domains, or Gene Ontology terms.

The tool allows viewing the genomic and transcriptomic data on multiple levels. For example, it contains pages for species (www.fungi.guru/species), genes (www.fungi.guru/sequence/view/20959), gene families (www.fungi.guru/family/view/117804), coexpression clusters (http://www.fungi.guru/cluster/view/741), neighborhoods (www.fungi.guru/network/graph/15360), phylogenetic trees of families (www.fungi.guru/tree/view/45802), Pfam domains (www.fungi.guru/interpro/view/5689), Gene Ontology terms (www.fungi.guru/go/view/16530) and others. These pages contain data relevant to the type of data displayed. For example, gene pages contain the functional annotation, the cDNA and protein sequences, assigned gene family, phylogenetic tree of the family, expression profiles, coexpression neighborhood and cluster, significantly similar neighborhoods, and Gene Ontology information. Conversely, Gene Ontology pages indicate the annotation of the GO term, the number of genes in the 19 fungi that have the GO term, and the co-expressed clusters that are enriched for the genes with this GO. A full description of the features is found at www.fungi.guru/features.

To exemplify these features, we provide three typical case studies addressing different aspects of fungal biology.

### 3.1 Example 1: identification of specialized metabolism hubs by coexpression analysis

**Supplementary figure 2:**
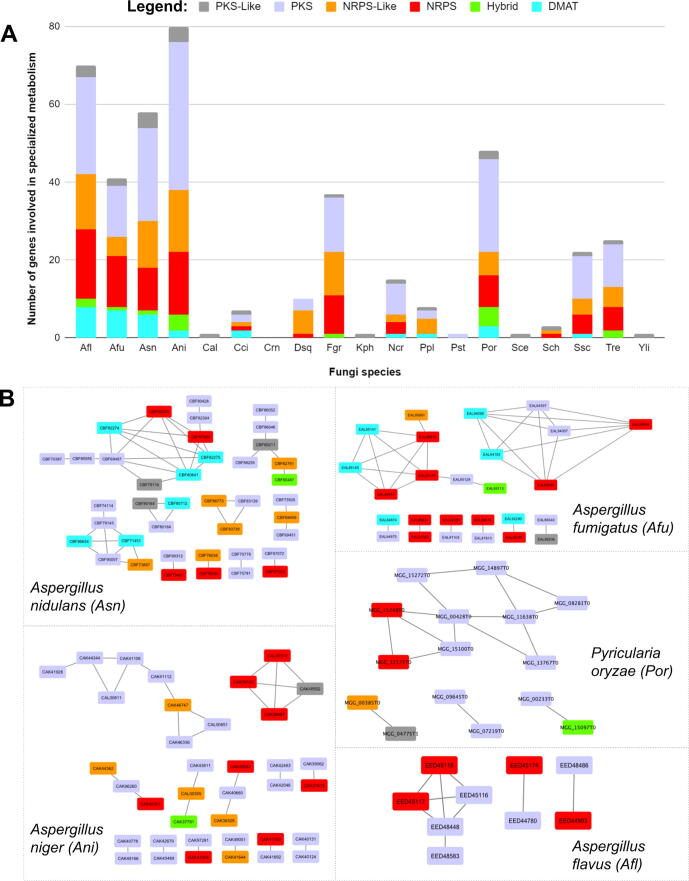


Coexpression analysis has been used successfully to study numerous metabolic pathways in plants [40], [41]. To exemplify how our tool can be used to study specialized metabolism in fungi, we first set to identify ‘backbone’ enzymes responsible for the biosynthesis of the different metabolite classes. To this end, we used the Secondary Metabolite Unique Regions Finder (SMURF) database [42], to assign the genes of the 19 fungi to nonribosomal peptide synthases (NRPSs), polyketide synthases (PKSs), NRPS-like enzymes, PKS-like enzymes, Hybrid enzymes and dimethylallyl tryptophan synthase (DMAT). The analysis revealed that the 19 fungi have a diverse set of the backbone enzymes, ranging from 80 enzymes in *Aspergillus niger* (Ani), to 0 in *Cryptococcus neoformans* (Crn), where five (Asn, Ani, Afu, Por, Afl, Table 1) of the 19 fungi were shown to have more than 40 backbone enzymes (Fig. S2A).

**Fig. 1:**
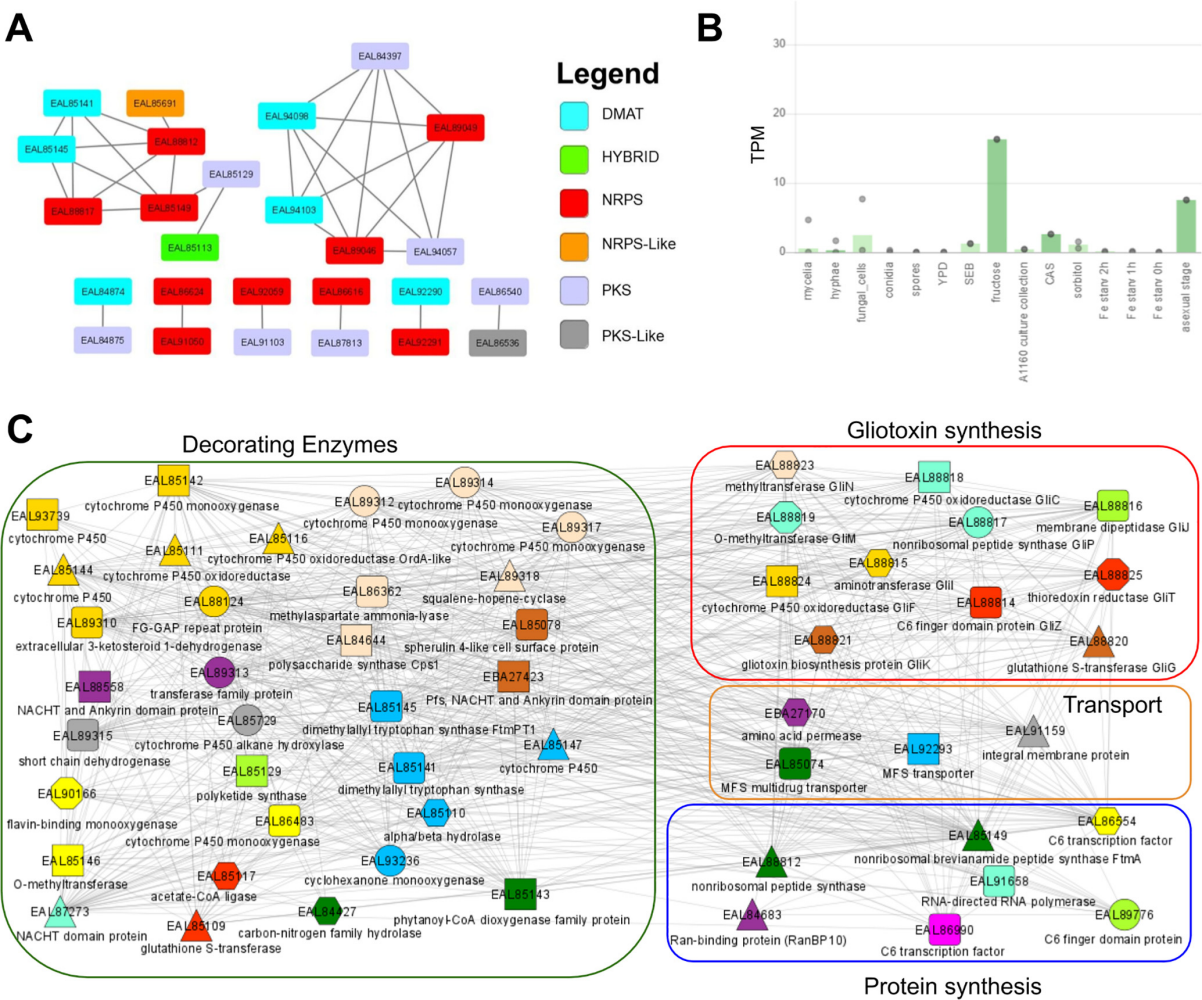
Coexpression analysis of specialized metabolic hubs in *Aspergillus fumigatus*. A) Coexpression network of the backbone enzymes involved in specialized metabolism. Nodes represent genes, while edges connect co-expressed genes. For brevity, enzymes that are not co-expressed are not shown. The color of the node indicates the type of backbone enzyme. The plot was generated by exporting the network file and opening the file in standalone Cytoscape. B) Expression profile of *EAL85149*, a nonribosomal peptide synthase ftmA that shows the highest connection to other backbone enzymes. The different samples are represented on the x-axis, while the gene expression values in the form of Transcripts Per Million (TPM) are indicated on the y-axis. The bars and the dots indicate the average and the minimum/maximum values of the RNA-seq experiments in the sample, respectively. C) Coexpression network of *EAL85149*. Nodes represent genes, while edges connect co-expressed genes. Colored shapes indicate which genes contain Pfam domains and orthogroups in common. For brevity, only part of the network is shown. The network contains four types of genes: gliotoxin family genes (indicated in the red rectangle), genes involved in protein synthesis (blue rectangle), metabolite transporters (orange rectangle), and decorating enzymes (green rectangle). The function of the genes can be inferred from the legend and by clicking on the nodes. For brevity, only the discussed genes are shown, and the nodes are arranged for better clarity. (For interpretation of the references to color in this figure legend, the reader is referred to the web version of this article.)

To identify groups of genes involved in metabolite synthesis and conditions that result in high metabolite production, backbone enzymes were entered into ‘Tools/Create custom network‘ tool (www.fungi.guru/custom_network/), which identifies coexpression relationships in the provided list of genes. To create the custom network, fungi.guru uses the existing, genome-wide co-expression network and extracts the specified nodes and edges. The custom network analysis of specialized metabolism genes in *Aspergillus fumigatus* produced an interactive network that revealed a high degree of coexpression among the backbone enzymes, indicating that biosynthesis of the various metabolites is transcriptionally coordinated (Fig. 1A).

One of the *Aspergillus fumigatus* backbone enzymes, *EAL85149*, nonribosomal peptide synthase *ftmA*, was found to be co-expressed with a large variety of genes involved in secondary metabolite synthesis (Fig. 1A), suggesting that the enzyme is part of a transcriptional program that produces numerous metabolites. By entering the EAL85149 into the search box, we arrived at a gene page dedicated to EAL85149 (www.fungi.guru/sequence/view/20959). The expression profile (www.fungi.guru/profile/view/27005) revealed that the gene shows the highest expression in fructose medium (Fig. 1B). Since specialized metabolism tends to be transcriptionally regulated [43], [44], this information can be used to suggest growth conditions needed to induce high production of specialized metabolites.

*EAL85149* is involved in the biosynthesis of toxins such as gliotoxins and brevianamide F, a precursor bioactive prenylated alkaloid [45]. Brevianamide F acts as a precursor for fungal toxins fumitremorgin C and trypacidin that can prevent phagocytosis of fungal conidia, and serves an essential protective role for the pathogenesis of fungi [46]. To identify genes that are functionally related to *EAL85149,* we viewed its coexpression network available at the gene page (www.fungi.guru/network/graph/15360) (Fig. 1C). 11 out of 13 gliotoxin biosynthesis genes are found in the gliotoxin gli coexpression cluster, with the exception of *GliA*, responsible for the exportation of extracellular gliotoxin essential for gliotoxin tolerance [47] and *GliH* whose function is still unknown [48]. Interestingly, the gliotoxin biosynthesis genes found in the coexpression cluster are also part of the genomic gli cluster, where the gli biosynthetic genes are found on the same chromosomal locus [49]. The coexpression cluster also contains a variety of transporters such as Major Facilitator Superfamily (MFS) transporter that could be used to secrete gliotoxin and other metabolites potentially synthesized by the coexpression cluster. Additionally, the coexpression cluster contains large numbers of cytochrome P450s, which are decorative enzymes essential for secondary metabolite synthesis [50].

**Supplementary figure 3:**
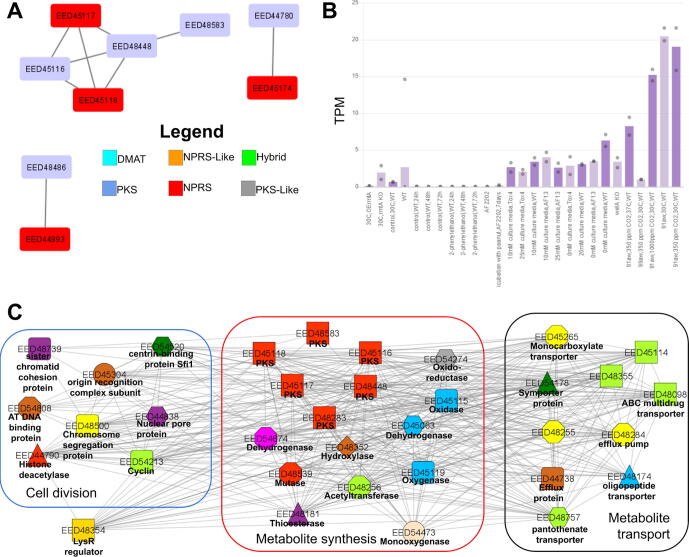


In addition to the discussed genes, we observed multiple genes coding for hypothetical proteins in the cluster. Since these genes are co-expressed with the gli pathway, they could also play a crucial role in secondary metabolite synthesis and merit further study. Thus, this example shows how fungi.guru can be easily used to reveal conditions that are likely to control the production of metabolites and reveal novel genes involved in the metabolite production (Fig. S3 shows a similar analysis performed in *Aspergillus flavus*).

### 3.2 Example 2: gene ontology search reveals clusters important for cellobiose degradation

Many fungi, especially saprobic fungi, obtain energy from decaying organic matter. Their main source of energy comes from digesting decomposing plant material such as cellulose and lignin [51]. Basidiomycetous fungi, for example, *Coprinopsis cinerea (Cci)* [52], are one of the most potent degraders of cellulose as they often grow in environments that are cellulose-abundant, such as dead wood and plants [53], [54]. Plant cell walls are multi-layered and made up of different materials which include cellulose, hemicelluloses, and lignins [55], leading to the need for fungi to produce various types of hydrolytic enzymes (endoglucanase, cellobiohydrolase, and beta-glucosidase) to break down these biomolecules into substances that can be used as sources of energy for cellular activity [56]. Thus, depending on the composition of the organic matter decomposed, different fungal species utilize their own unique set of enzymes and metabolic pathways for cellobiose degradation [57].

**Fig. 2:**
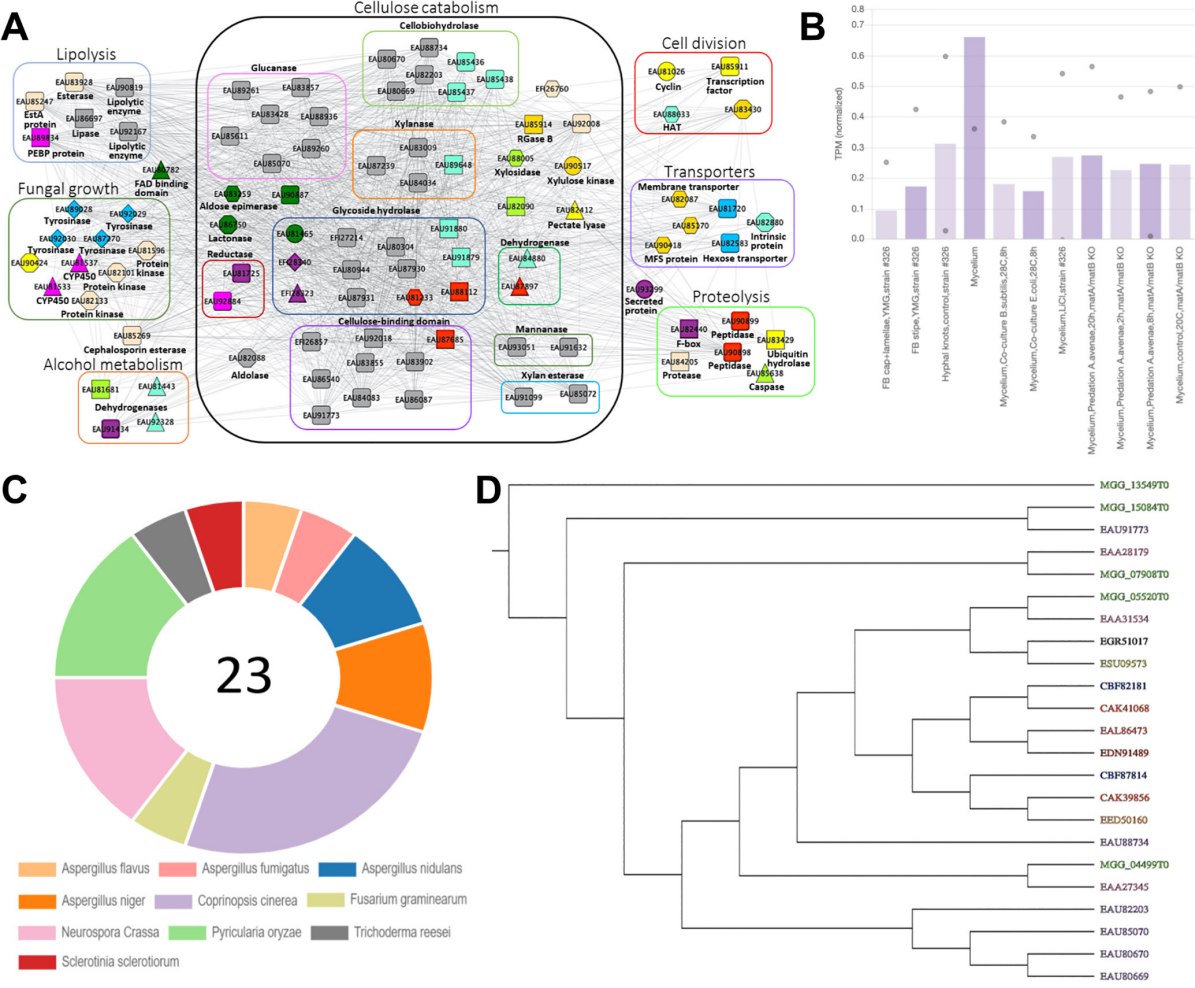
Cluster analysis involved in cellulose catabolic process in *Coprinopsis cinerea*. A) Coexpression network of genes identified as enriched for GO:0030245 (GO term for Cellulose Catabolic Process). Nodes represent genes, while co-expressed genes are connected by edges. For brevity, novel genes (hypothetical proteins) are not shown in the network. Colored shapes indicate which genes contain Pfam domains and orthogroups in common. B) Average expression profile of cluster 21 for *Cci*. Only part of the expression profile containing the discussed sample is shown. The different samples are represented on the x-axis, while the gene expression values in the form of Transcripts Per Million (TPM) are indicated on the y-axis. The bars and the dots indicate the average and minimum/maximum values of the RNA-seq experiments in the sample. C) The proportion of sequences in the cellobiohydrolases Gene Family: OG_03_0001111. There are a total of 23 sequences with this label, from 10 different species. D) Phylogenetic tree of cellobiohydrolases gene family OG_03_0001111.

To gain insight into cellobiose degradation in *Cci*, we entered ‘cellulose catabolic process’ into ‘Tools/Find enriched clusters’ (www.fungi.guru/search/enriched/clusters), which revealed Gene Ontology term GO:0030245 (GO term for Cellulose Catabolic Process). The tool identified six clusters significantly enriched (p-value for enrichment < 0.05) for this GO process in six species (*Aspergillus nidulans*, *Aspergillus niger*, *Coprinopsis cinerea*, *Neurospora crassa*, *Pyricularia oryzae,* and *Sclerotinia sclerotiorum*). For *Cci*, we focused on cluster 21, which was enriched for GO:0030245 (p-value for enrichment < 0.001). Using the tool, we arrived at a page that details the cluster analysis of genes involved in cellobiose degradation in *Cci* (www.fungi.guru/cluster/view/741) (Fig. 2A).

The coexpression cluster contained 150 genes, which were involved in different processes (alcohol metabolism, cellulose catabolism, cell division, fungal growth, lipolysis, proteolysis, and transporters) (Fig. 2A). We observed that cellobiohydrolases [58], cellulose-binding domains [59], glucanases [60], glycoside hydrolases [61], xylanases [62], and sugar transporters are the main genes present in the cluster, supporting the function of this cluster in cellobiose degradation and uptake of sugars. The cluster page also contains information about the enriched GO terms, whether similar clusters can be found in other fungi, presence of InterPro domains, enriched GO terms, and presence of gene families. Additionally, we also investigated the expression profile for this cluster (Fig. 2B) and observed that the gene expression is the highest in the mycelium, suggesting that these structures are most active in degrading cellulose in *Cci*.

The most abundant gene family in cluster 21 was OG_03_0001111, which contains genes annotated as cellobiohydrolases. We clicked on OG_03_0001111 to arrive at a page dedicated to the family (www.fungi.guru/family/view/117804). The gene family page revealed that *Cci* contains the highest proportion of cellobiohydrolases (Fig. 2C), suggesting that it is one of the more potent degraders of cellobiose and its cellobiohydrolase gene family may have undergone a higher degree of expansion compared to the other species. The page also contains a link to the phylogenetic tree generated by Orthofinder (www.fungi.guru/tree/view/45802). The tree (Fig. 2D) shows us that the cellobiohydrolase family has expanded in *Cci* and this could hypothetically be attributed to the further enhancement of cellobiose degradation ability in *Cci*.

Since we identified multiple such clusters in different fungi (please see a similar analysis done in *Postia placenta*, Fig. S4), we propose that our tool is a good starting point to study cellulose degradation in fungi. Thus, the tool allows easy identification of coexpression clusters, genes, and gene families relevant to the biological process of interest.

### 3.3 Example 3. Comparative analyses of DNA replication in fungi

Coexpression relationships can be conserved across species, where coexpression clusters containing the same gene families and Pfam domains are found in multiple species [17], [18], [63]. Identifying these conserved coexpression clusters thus enables the identification of conserved biological pathways and the core genetic components of these pathways [16], [18], [63], [64]. Fungi.guru contains multiple tools that allow the identification of the conserved pathways.

**Fig. 3:**
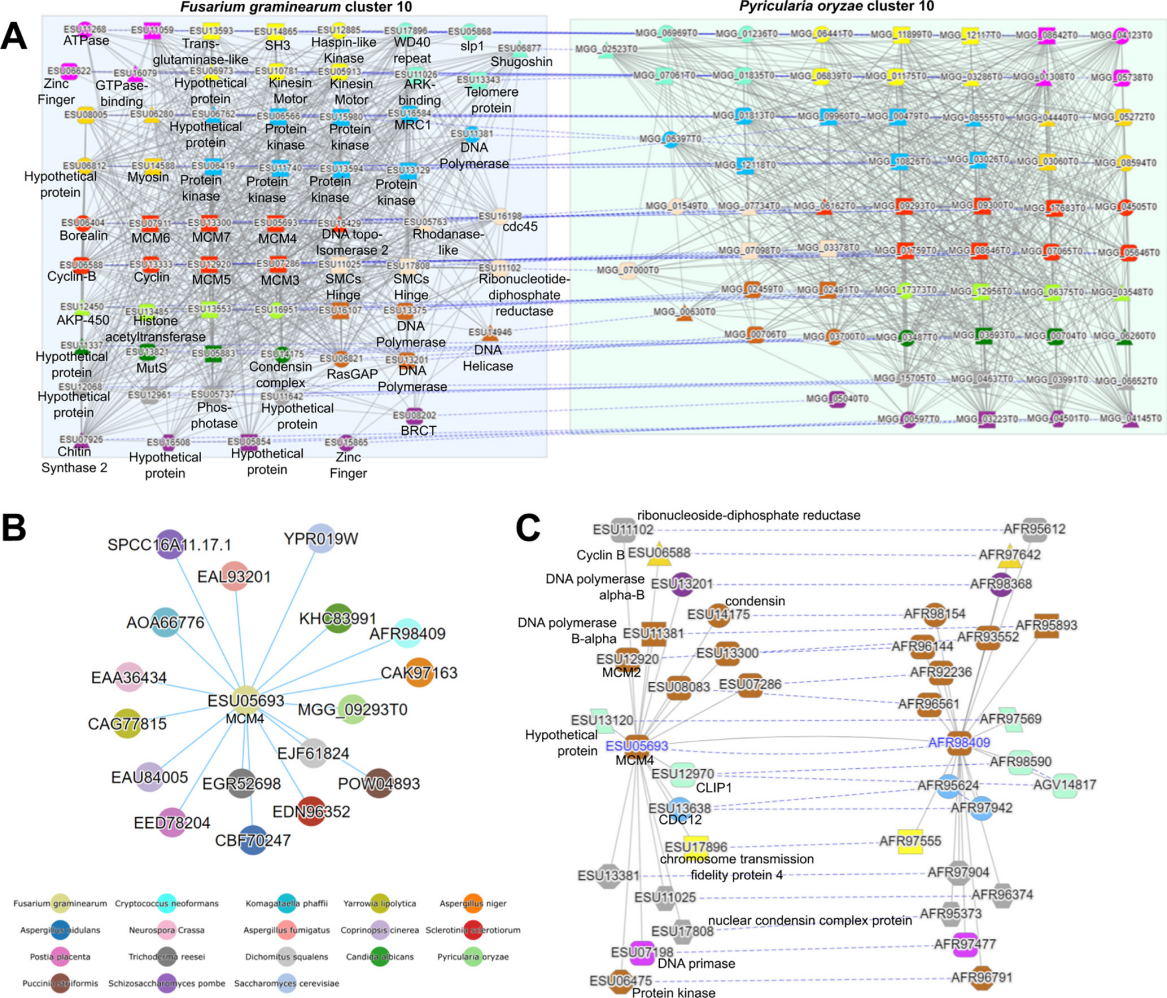
Comparative analysis of gene coexpression networks of DNA replication in *F. graminearum* and *P. oryzae*. A) Comparative analysis of conserved coexpression clusters. Nodes represent genes, while co-expressed genes are connected by edges. The color and shape of the nodes indicates the gene family and pfam domains of the genes. The dashed edges between the conserved clusters connect genes that belong to the same gene families, while gray edges connect co-expressed genes. B) Expression context conservation network of *ESU0569.* Each node indicates a gene co-expression neighborhood, while blue edges connect conserved neighborhoods. The color of the nodes indicates the fungal species. C) Conserved co-expression neighborhoods. Please see text in panel 3A for legend. (For interpretation of the references to color in this figure legend, the reader is referred to the web version of this article.)

To exemplify these comparative features, we examined the process of DNA replication between 2 fungi known for their infectious capabilities - *Fusarium graminearum* and *Pyricularia oryzae*. Under ‘Tools/Find enriched clusters’, we searched for enriched clusters using the GO term “DNA replication” with *F. graminearum* selected as target (www.fungi.guru/search/enriched/clusters). By clicking “Show cluster”, the tool revealed that Cluster_10 in *F. graminearum* is enriched for this term (www.fungi.guru/cluster/view/1203). Under ‘Similar Clusters’ table, we identified *P. oryzae* cluster 10 as being similar to *F. graminearum* cluster 10 with Jaccard index value of 0.202 [34]. Clicking on the ‘Compare’ button shows the coexpression networks of these two conserved modules, where only genes that have a homolog or Pfam domain in both clusters are shown (Fig. 3A).

As expected for clusters significantly enriched for GO term DNA replication, the genes in the conserved clusters are involved in chromatin regulation and DNA replication. The genes belong to multiple conserved gene families, including histone acetyltransferase, minichromosome maintenance protein complex (MCM), DNA polymerase, and others (Fig. 3A). Histone acetyltransferases are involved in the loosening of the condensed chromatin structure to allow DNA replication machinery access to the template [65]. Cyclin-B activates downstream cyclin-dependent kinases involved in the M phase of the cell cycle, while other cyclin types control kinases for other stages in the cell cycle [66]. MCM is known to be involved in the assembly of the replication initiation complex at the origin of replication [67]. Cdc45 form the catalytic core of DNA helicase that serves to unwind the double-stranded DNA [68]. Type 2 DNA topoisomerase introduce negative supercoils in the DNA to release the stress on the DNA set by the unwinding action of DNA Helicase [69].

In addition to identifying conserved clusters, our tool can also reveal which gene coexpression neighborhoods are similar. For example, *ESU05693* (www.fungi.guru/sequence/view/89556) is a DNA replication licensing factor mcm4 that belongs to OG_03_0001507 gene family (www.fungi.guru/family/view/118200). The gene page for *ESU05693* contains a table ‘Expression context conservation’ which lists other genes from OG_03_0001507 gene family that have a coexpression neighborhood that contains similar content of gene families to *ESU05693*. By clicking on ‘View ECC as graph’, the tool indicated that 17 genes from the OG_03_0001507 gene family belonging to 17 different species have similar coexpression neighborhoods (Fig. 3B). This is not surprising as DNA replication is a well-conserved process. The contents of the co-expressed neighborhoods can be viewed by clicking on the ‘View ECC pair as graph’ in the ‘Expression context conservation’ table. Similarly to the comparative analysis of the clusters (Fig. 3A), the tool displays the contents of the two neighborhoods and highlights the conserved gene families and Pfam domains (Fig. 3C). The contents of the two coexpression neighborhoods are also related to DNA replication. Thus, fungi.guru allows identifying conserved coexpression clusters and neighborhoods across species, which can be invaluable to identify novel genes and pathways relevant to a biological pathway of interest. Since the genes in these conserved clusters belong to the same orthogroups and show similar co-expression patterns across species, the conserved clusters can be used to identify functional orthologs.

## 4 Conclusion

To help remedy the paucity of functional information for fungi, we constructed www.fungi.guru, a user-friendly online tool that facilitates the analysis of genomic and transcriptomic data. The available tools allow for the identification and analysis of co-expressed gene neighborhoods, clusters, and gene families. While our examples addressed specialized metabolism, cellulose degradation, and cell division, fungi.guru can be used to study molecular function, biological process, and cellular component of all genes expressed in the 19 fungi. We envision that the gene function prediction tools present in fungi.guru will enhance the selection of relevant genes to further functional analyses.

## 5 Data availability

The expression matrices, RNA-seq sample annotation and gene families are available from the Supplementary Data. The co-expression networks, coding and protein sequences can be downloaded from www.fungi.guru.

## Declaration of Competing Interest

None of the authors have any competing interests.

The data is available from www.fungi.guru and from the supplemental material.

## Figures and Tables

**Supplementary figure 4:**